# Differential Membrane Dipolar Orientation Induced by Acute and Chronic Cholesterol Depletion

**DOI:** 10.1038/s41598-017-04769-4

**Published:** 2017-06-30

**Authors:** Parijat Sarkar, Hirak Chakraborty, Amitabha Chattopadhyay

**Affiliations:** 1 CSIR-Centre for Cellular and Molecular Biology, Uppal Road, Hyderabad, 500 007 India; 2 School of Chemistry, Sambalpur University, Jyoti Vihar, Burla, Odisha 768 019 India

## Abstract

Cholesterol plays a crucial role in cell membrane organization, dynamics and function. Depletion of cholesterol represents a popular approach to explore cholesterol-sensitivity of membrane proteins. An emerging body of literature shows that the consequence of membrane cholesterol depletion often depends on the *actual* process (acute or chronic), although the molecular mechanism underlying the difference is not clear. Acute depletion, using cyclodextrin-type carriers, is faster relative to chronic depletion, in which inhibitors of cholesterol biosynthesis are used. With the overall goal of addressing molecular differences underlying these processes, we monitored membrane dipole potential under conditions of acute and chronic cholesterol depletion in CHO-K1 cells, using a voltage-sensitive fluorescent dye in dual wavelength ratiometric mode. Our results show that the observed membrane dipole potential exhibits difference under acute and chronic cholesterol depletion conditions, *even when cholesterol content was identical*. To the best of our knowledge, these results provide, for the first time, molecular insight highlighting differences in dipolar reorganization in these processes. A comprehensive understanding of processes in which membrane cholesterol gets modulated would provide novel insight in its interaction with membrane proteins and receptors, thereby allowing us to understand the role of cholesterol in cellular physiology associated with health and disease.

## Introduction

Biological membranes are complex, non-covalent, highly organized, two-dimensional assemblies of a diverse variety of lipids and proteins that allow confinement of intracellular contents in selective compartments. They impart an identity to individual cells and organelles, besides providing an appropriate environment for proper functioning of membrane proteins. A major representative lipid in higher eukaryotic cellular membranes is cholesterol which is the end product of a long and multistep sterol biosynthetic pathway that parallels sterol evolution^1, 2^. Understanding the role of cholesterol is important to gain insight into membrane structure, function, organization and dynamics^3–5^. This is evident from the range of effects it exerts on membranes such as modulation of membrane order, extent of water penetration and membrane thickness^6–9^. Cholesterol is often found distributed nonrandomly in domains or pools in biological and model membranes^3, 10–14^. Many of these domains (sometimes termed as ‘lipid rafts’) are believed to be important for the maintenance of membrane structure and function. The idea of such specialized membrane domains assumes significance in cell biology since many physiologically important functions such as membrane sorting and trafficking^15^, signal transduction processes^16^, in addition to the entry of pathogens^17, 18^ have been attributed to these domains.

Dipole potential is an important electrostatic property of organized molecular assemblies (such as membranes and micelles). The origin of dipole potential is the electrostatic potential difference within the assembly due to the nonrandom arrangement of amphiphile dipoles and solvent (water) molecules at the assembly interface^19–24^. Dipole potential has received relatively less attention in the literature as opposed to transmembrane and zeta potential, and its role in membrane protein function^25^ has not been comprehensively addressed. Depending on the orientation of electric dipoles at the membrane interface, the magnitude of dipole potential has been estimated to be 200–1000 mV. Since dipole potential is operative over a relatively small distance within the membrane, the electric field generated due to dipole potential could be very large (~10^8^–10^9^ Vm^−1^)^20–23^.

In this work, we have explored the possible correlation between cell membrane cholesterol content and membrane dipole potential, under conditions of acute (*e.g*., by using carriers such as methyl-β-cyclodextrin (MβCD)) and chronic (metabolic depletion using cholesterol biosynthetic inhibitors) cholesterol depletion. In order to understand the mechanistic framework of membrane organization accompanying modulation of membrane cholesterol, we carried out dipole potential measurements of CHO-K1 cells by a dual wavelength ratiometric imaging approach using an electrochromic probe di-8-ANEPPS^26–30^. Interestingly, membrane cholesterol has been shown to increase dipole potential in model and natural membranes^25, 29, 30^ in a stereo-specific manner^31^. In spite of these important structural correlates, the molecular mechanism underlying the modulation of membrane cholesterol is not clear, particularly with reference to the manner in which depletion is carried out (acute *vs*. chronic). We show here, by measurement of membrane dipole potential, that dipolar reorganization could be very different in acute and chronic cholesterol depletion, even when the extent of cholesterol depletion is identical.

## Results

### Concentration-dependent cholesterol depletion from cell membranes by MβCD

**Figure 1:**
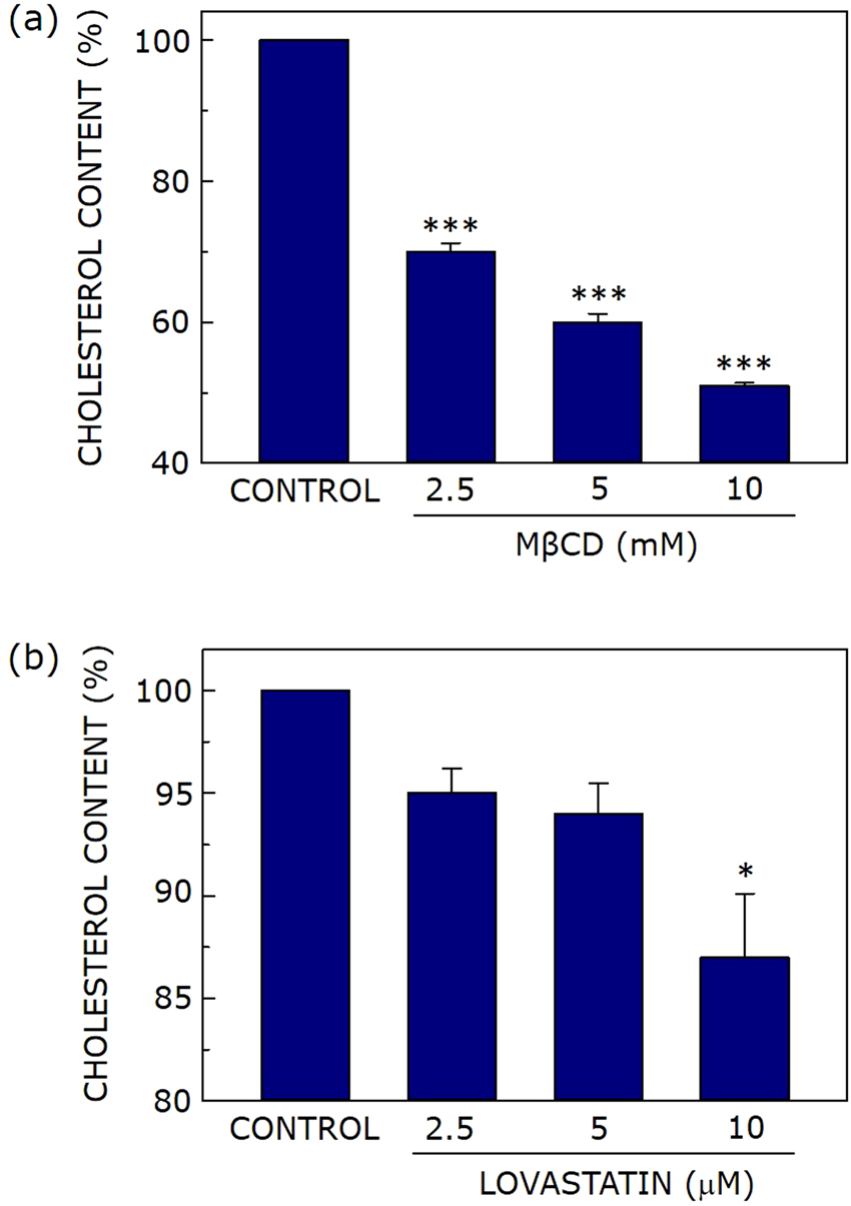
Membrane cholesterol depletion upon acute or chronic treatment. Effect of increasing concentration of (**a**) MβCD and (**b**) lovastatin on cholesterol content of CHO-K1 cell membranes. Values are expressed as percentage of cholesterol content for cell membranes in the absence of MβCD or lovastatin treatment. Data represent means ± S.E. of at least three independent measurements (* and *** correspond to significant (*p* < 0.05 and *p* < 0.001) difference in cholesterol content of MβCD or lovatstatin-treated cell membranes relative to untreated membranes). See Methods for other details.

Modulation of membrane cholesterol has proved to be an important tool to address cholesterol-dependent function of membrane proteins. For example, we have previously shown that membrane cholesterol is required for the organization and function of the serotonin_1A_ receptor, an important member of the G protein-coupled receptor family (GPCR)^32, 33^. This was shown by the depletion of membrane cholesterol either in an acute^34, 35^ or chronic^36, 37^ manner. Acute cholesterol depletion is achieved by physical depletion of cholesterol using carriers such as MβCD, a water soluble carbohydrate polymer that can selectively and efficiently extract cholesterol from membranes by including it in a central nonpolar cavity^38, 39^. Figure 1a shows cholesterol content in membranes of cholesterol-depleted CHO-K1 cells. Upon treatment with increasing concentration of MβCD, the cholesterol content of cell membranes shows progressive reduction. For example, cholesterol content was reduced to ~70% of control (without treatment) upon treatment of membranes with 2.5 mM MβCD. Maximum (~50%) reduction in cholesterol content was achieved with 10 mM MβCD (see Fig. 1a). The concentration range of MβCD was carefully chosen to minimize replenishment of membrane cholesterol during the experiment and to avoid loss of phospholipids. The change in phospholipid content under these conditions was negligible, even when 10 mM MβCD was used (see Fig. S1).

### Chronic cholesterol depletion upon statin treatment

Membrane cholesterol depletion using MβCD suffers from a number of limitations^38, 40^. A major limitation is that cholesterol depletion using MβCD is an acute process due to the relatively short time of treatment. Acute cholesterol depletion therefore may not be a faithful indicator of physiologically relevant cholesterol modulation due to its short time scale. On the other hand, metabolic (chronic) depletion of cholesterol is typically achieved using inhibitors of cholesterol biosynthesis such as statins. Statins are a class of molecules that act as competitive inhibitors of HMG-CoA reductase, the rate-limiting key enzyme in the cholesterol biosynthetic pathway^41, 42^. Statins are top selling drugs globally and in clinical history^43^. They are extensively used as oral cholesterol lowering drugs to treat hypercholesterolemia and dyslipidemia^41^. Lovastatin is a commonly used statin which lowers cholesterol content by inhibiting HMG-CoA reductase. Since cholesterol lowering (depletion) by statins takes place over a relatively long period of time, it represents a chronic process, and is physiologically more relevant^37, 44^. Figure 1b shows that upon treatment of CHO-K1 cells with increasing concentrations of lovastatin, the cell membrane exhibits progressive reduction in cholesterol content, and ~13% of cholesterol is metabolically depleted when 10 μM lovastatin was used. Control experiments using MTT assay showed that cell viability remained invariant under conditions of acute and chronic cholesterol depletion (data not shown).

### Cholesterol depletion reduces dipole potential of cell membranes

**Figure 2:**
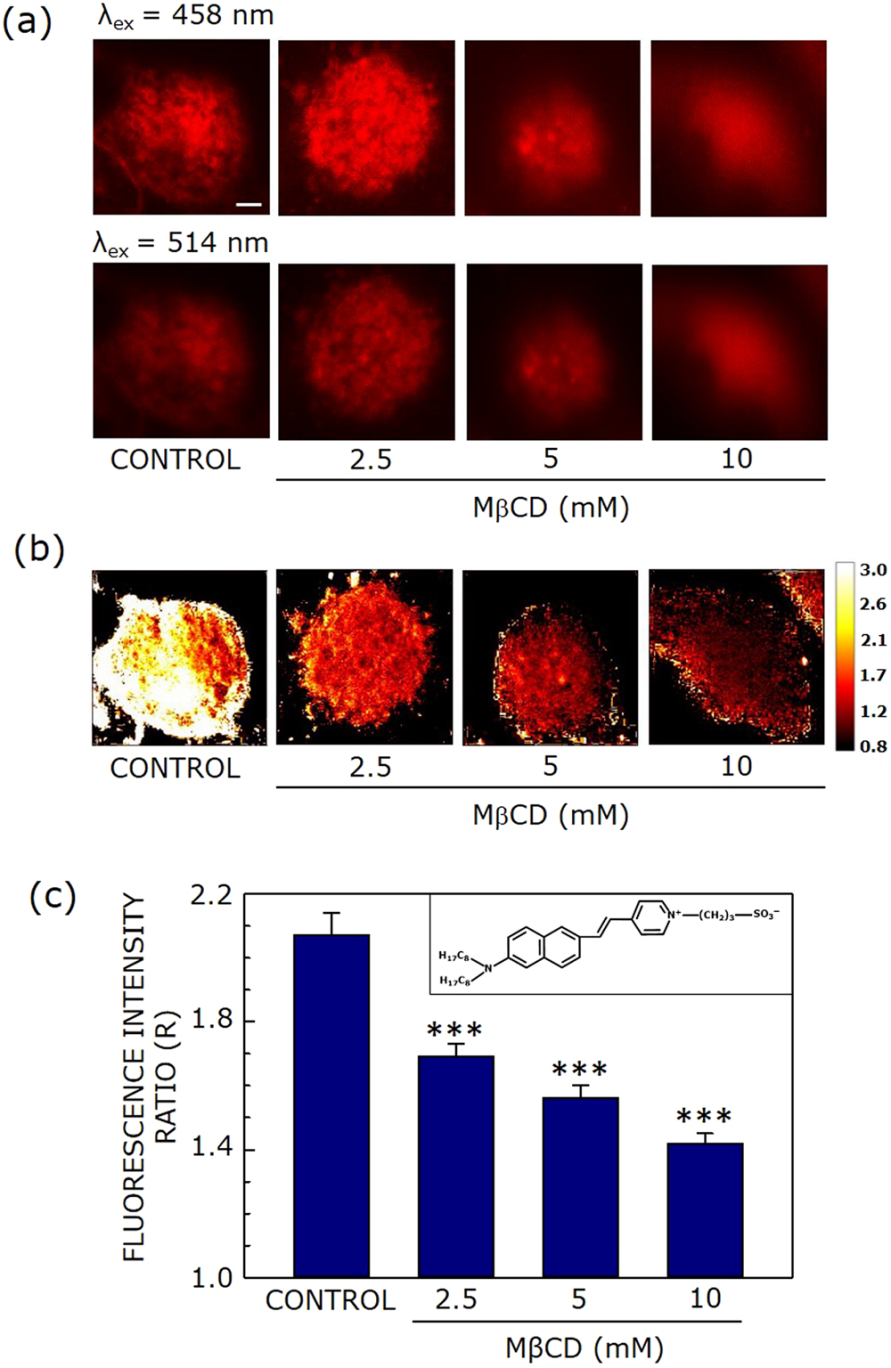
Effect of acute cholesterol depletion on membrane dipole potential. (**a**) Representative confocal micrographs of CHO-K1 cells labeled with di-8-ANEPPS (λ_ex_ = 458 nm (upper panel), and λ_ex_ = 514 nm (lower panel), the emission band pass being 650–710 nm in both cases) with increasing concentrations of MβCD. Fluorescence intensity of images was corrected for laser power at two different excitation wavelengths (458 and 514 nm). (**b**) The corresponding fluorescence intensity ratio (R) map (color coded in a scale of 0.8–3) under these conditions. R is defined as the ratio of fluorescence intensities at an excitation wavelength of 458 nm to that at 514 nm (emission band pass at 650–710 nm in both cases) and was calculated using ImageJ (NIH, Bethesda, MD). (**c**) Effect of increasing concentration of MβCD on the R value (means ± S.E.), averaged over at least fifteen independent measurements (*** corresponds to significant (*p* < 0.001) difference in R). The inset shows the chemical structure of di-8-ANEPPS. The scale bar indicates 10 μm. See Methods for more details.

We utilized the voltage-sensitive fluorescent probe di-8-ANEPPS (see inset in Fig. 2c) for estimating dipole potential of control and cholesterol-depleted cell membranes. The underlying mechanism for the voltage sensitivity of this probe is believed to be electrochromic in nature, leading to a shift of its excitation spectrum, and the shift is proportional to the local electric field strength^45, 46^. The advantage of using di-8-ANEPPS is that it undergoes very slow internalization^47^. As a result, the entire fluorescence of di-8-ANEPPS originates from the cell membrane.

**Figure 3:**
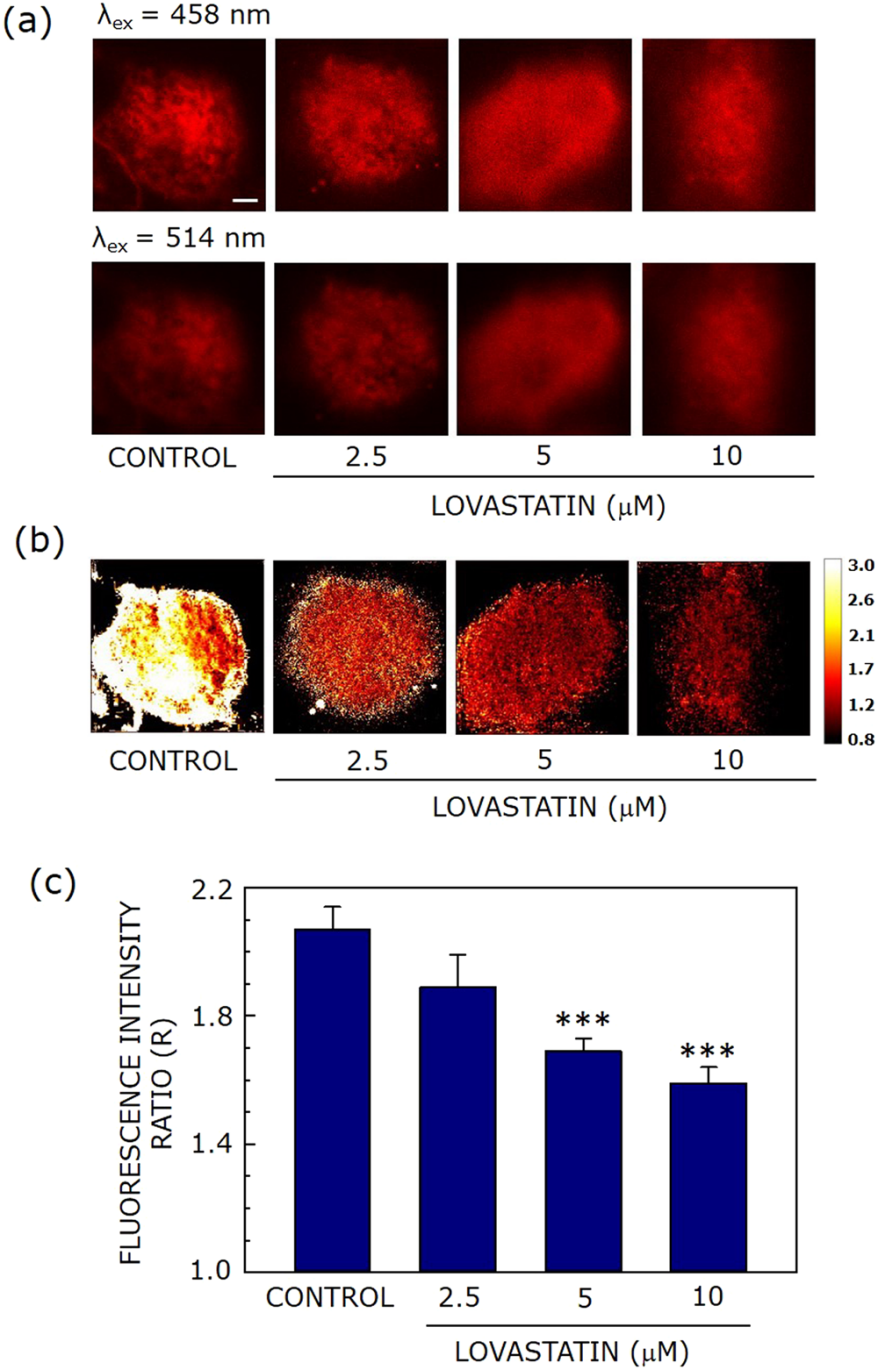
Effect of chronic cholesterol depletion on membrane dipole potential. Representative confocal micrographs of CHO-K1 cells labeled with di-8-ANEPPS (λ_ex_ = 458 nm (upper panel), and λ_ex_ = 514 nm (lower panel), the emission band pass being 650–710 nm in both cases) with increasing concentrations of lovastatin. Fluorescence intensity of images was corrected for laser power at two different excitation wavelengths (458 and 514 nm). (**b**) The corresponding fluorescence intensity ratio (R) map (color coded in a scale of 0.8–3) under these conditions. R is defined as the ratio of fluorescence intensities at an excitation wavelength of 458 nm to that at 514 nm (emission band pass at 650–710 nm in both cases) and was calculated using ImageJ (NIH, Bethesda, MD). (**c**) Effect of increasing lovastatin concentration on the R value (means ± S.E.), averaged over at least fifteen independent measurements (*** corresponds to significant (*p* < 0.001) difference in R). The scale bar indicates 10 μm. See Methods for more details.

Figures 2a and 3a show representative confocal micrographs of di-8-ANEPPS- labeled CHO-K1 cells using two excitation wavelengths (458 and 514 nm, upper and lower panels, respectively) and emission collected using a 650–710 nm band pass (for both excitation wavelengths), with increasing concentrations of MβCD or lovastatin, respectively. The useful parameter in this method of dipole potential measurement is the fluorescence ratio (R) which is the ratio of fluorescence intensities at two different excitation wavelengths with emission wavelength being fixed at the same wavelength. This ratio is sensitive to any change in the dipolar field where the potential-sensitive probe di-8-ANEPPS is localized, and is independent of specific molecular interactions^26, 48^. Figure 2b shows a representative map of R (calculated from the ratio of fluorescence intensities from two panels in Fig. 2a) of CHO-K1 cell membranes under conditions of acute cholesterol depletion using increasing concentration of MβCD. Figure 2b shows that there is progressive reduction in R with increasing MβCD concentration, *i.e*., with decreasing membrane cholesterol. To obtain a quantitative estimate of R, averaged over a large number of cells, we plotted R (averaged over fifteen different fields) with increasing MβCD concentration (see Fig. 2c). The figure shows progressive decrease in R with increasing cholesterol depletion, in overall agreement with Fig. 2b. This is in agreement with previous work by us^25, 30^ and others^29^ where cholesterol was shown to increase dipole potential in membranes. Figure 2c shows ~33% reduction in R in cell membranes treated with 10 mM MβCD (corresponding to a ~50% reduction in membrane cholesterol, see Fig. 1a).

Figure 3 shows the corresponding change in dipole potential (R) under conditions of chronic cholesterol depletion using lovastatin. The change in R upon chronic depletion (with increasing concentration of lovastatin) is shown in Fig. 3b (in a chosen field) and Fig. 3c (averaged over fifteen fields). The decrease in R under these conditions was ~24% relative to control membranes. Interestingly, we observed that the change in membrane dipole potential upon cholesterol depletion was reversed upon replenishment with cholesterol (see Figs S2 and S3).

### Differential membrane organization revealed by change in dipole potential under acute and chronic cholesterol depletion

Work from a number of laboratories have demonstrated the crucial role of membrane cholesterol in the function of membrane proteins and receptors^32, 33, 49–55^. However, the detailed mechanism underlying the effect of membrane cholesterol on the structure and function of membrane proteins appears complex^56, 57^. A popular approach to explore cholesterol-sensistivity of membrane proteins has been depletion of membrane cholesterol, followed by monitoring membrane protein function. As mentioned above, this can be achieved by acute^34, 35, 58–63^ or chronic^36, 37, 44, 63–65^ depletion of membrane cholesterol. Interestingly, the consequences of acute and chronic cholesterol depletion are often different^66–72^ and this has resulted in a discussion on the molecular mechanism underlying these processes. For example, these two processes (acute and chronic depletion) have very different consequences on the organization of GPI-anchored proteins and caveole^66^, activity of Na-P_i_ cotransporter^69^, and induction of autophagy^71^. In addition, membrane dynamics (lateral diffusion) appears to be differentially modulated, depending on the actual process of cholesterol depletion^67, 68, 70^. We have previously shown that the function^34, 37^ and oligomerization^72^ of GPCRs such as the serotonin_1A_ receptor exhibit differential response to the actual process of cholesterol modulation.

A closer inspection reveals that the process of cholesterol depletion by agents such as MβCD differs significantly from chronic cholesterol depletion using cholesterol biosynthetic inhibitors such as lovastatin. A hallmark of membrane cholesterol is its nonrandom distribution in domains (or pools) in biological and model membranes^13, 14, 73, 74^. Cholesterol depletion using carriers such as MβCD is known to be a multiphasic process, characterized by differential efficiency of extracting cholesterol from various membrane domains^38, 75, 76^. There appears to be little consensus regarding (differential) extraction efficiency of agents such as MβCD in relation to domain organization of membrane cholesterol and specific experimental conditions used play an important role^39, 66, 76–78^. Although it has been recently reported that acute cholesterol depletion results in loss of cholesterol preferentially from liquid-disordered regions in model membranes^77, 78^, this does not appear to be true in the complex and heterogeneous cellular environment where MβCD does not appear to extract cholesterol preferentially from any specific type of membrane fraction (domain)^79^. On the other hand, chronic cholesterol depletion using biosynthetic inhibitors of cholesterol works in a completely different manner by simply reducing the cellular production of cholesterol, prior to cholesterol localization in various membrane domains. However, chronic cholesterol depletion is complicated by the fact that the effects could be pleiotropic in nature^80^ and could even induce cell cycle arrest^81^. In the backdrop of this overall scenario, we addressed fundamental molecular level difference between these two processes by measurement of membrane dipole potential.

**Figure 4:**
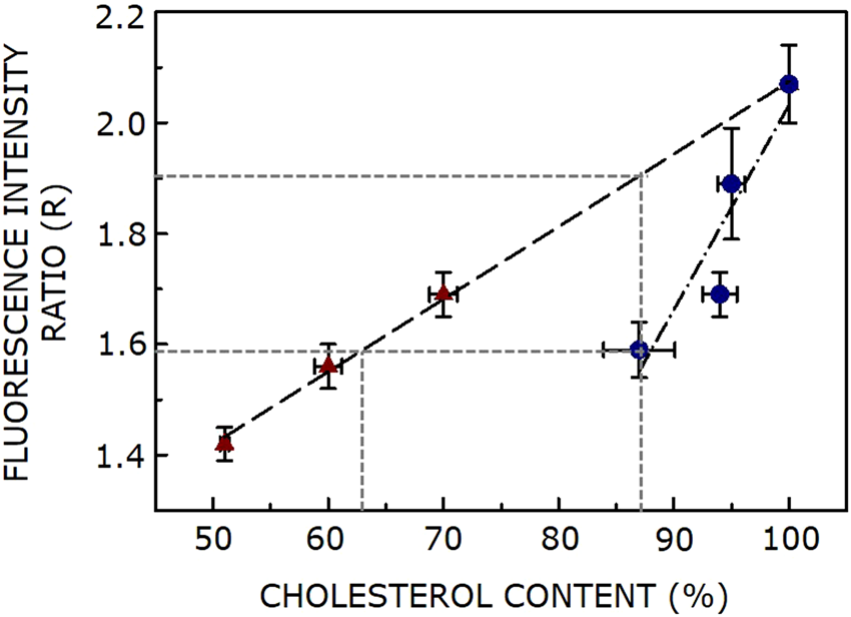
Differential dependence of membrane dipole potential on membrane cholesterol content under acute and chronic depletion conditions. A plot of R (a measure of membrane dipole potential, values taken from Figs 2c and 3c) with membrane cholesterol content for acute (MβCD; maroon triangle) and chronic (lovastatin; blue circle) depletion of cholesterol. The cholesterol content data is from Fig. 1. Data points represent means ± S.E. and the lines shown are linear fits. An interesting feature is the difference in slope observed under acute (~0.013) and chronic (~0.037) depletion conditions, thereby indicating a stronger dependence of R on cholesterol content under chronic depletion condition. The orthogonal projections on the axes show that *membrane dipole potential could vary appreciably even when membrane cholesterol content is identical*. See text for more details.

In order to gain insight into cholesterol-dependent changes in terms of membrane dipole potential, we plotted R (a measure of dipole potential, from Figs 2c and 3c) as a function of membrane cholesterol content following cholesterol depletion under these conditions (from Fig. 1). This plot is shown in Fig. 4. The figure shows that R drops linearly with decreasing membrane cholesterol content in both cases. A striking feature is the difference in slope observed under acute (~0.013) and chronic (~0.037) depletion conditions, with ~2-fold higher slope when cholesterol was depleted in a chronic fashion. This indicates stronger dependence of R on cholesterol content under chronic depletion condition, implying thereby that there is an intrinsic difference between these two processes. When highest concentration (10 μM) of lovastatin was used, ~87% of cholesterol was retained, corresponding to R of ~1.6. A careful inspection of the figure shows that at identical R, ~63% cholesterol remained when depletion was carried out in an acute manner. While it has been previously reported that increasing membrane cholesterol results in higher membrane dipole potential^25, 29, 30^, our present work shows that membrane dipole potential could depend on the actual process used to deplete cholesterol, and not on absolute cholesterol content in the membrane. To the best of our knowledge, these results provide, for the first time, difference between acute and chronic cholesterol depletion at the molecular level in terms of membrane dipolar reorientation.

## Discussion

A fundamental difference between chronic and acute cholesterol depletion is the kinetics of the process. Chronic depletion is a relatively slow process and therefore there is enough time for membrane reorganization, even allowing some of the slowest steps to take place. This will have an effect on membrane dipole potential. Acute depletion, on the other hand, is a faster process and membrane reorganization may not be complete under these conditions. The fact that results from acute cholesterol depletion varies tremendously with experimental conditions (time of treatment, concentration of MβCD and cell type)^38^ further supports this proposition.

Work from a number of groups has shown that membrane dipole potential is a sensitive indicator of the nature of membrane lipid^29–31, 82^, the function of membrane proteins and peptides^25, 28, 83–85^ and in monitoring lipid-protein interaction^86, 87^. Interestingly, we recently introduced the concept of membrane dipole potential in case of organized molecular assemblies such as micelles and showed that micellar dipole potential is a reliable indicator of the process of micellization and shape transition^24, 88^. In this work, we provide a simple and straightforward method to measure dipole potential of cell membranes using commercially available fluorescence confocal microscopic set-up. Our results show that membrane dipole potential, in addition to be dependent on membrane cholesterol content, could reveal interesting difference in dipolar reorientation of membrane components (possibly due to differential membrane reorganization) induced by acute and chronic cholesterol depletion. We believe that these results provide novel insight at a molecular level in the complex interplay between cholesterol and membrane proteins which gets manifested in a variety of biological phenomena. In addition, this could be useful in future drug design since statins represent the best selling drugs in clinical history^43, 89^.

## Methods

### Materials

1,2-dimyristoyl-*sn*-glycero-3-phosphocholine (DMPC), CaCl_2_, EDTA, gentamycin sulfate, MβCD, 3-(4,5-dimethylthiazol-2-yl)−2,5-diphenyl-tetrazolium bromide (MTT), MgCl_2_, Na_2_HPO_4_, penicillin, phenylmethylsulfonyl fluoride (PMSF), sodium bicarbonate, streptomycin, and Tris were obtained from Sigma Chemical Co. (St. Louis, MO). DMEM/F-12 [Dulbecco’s modified Eagle’s medium/nutrient mixture F-12 (Ham) (1:1)] and fetal calf serum (FCS) were from Gibco/Life Technologies (Grand Island, NY). Bicinchoninic acid (BCA) reagent for protein estimation was from Pierce (Rockford, IL). Lovastatin was obtained from Calbiochem (San Diego, CA). 4-(2-(6-(Dioctylamino)-2-naphthalenyl)ethenyl)-1-(3-sulfopropyl)-pyridinium inner salt (di-8-ANEPPS) was purchased from Molecular Probes (Eugene, OR). The concentration of stock solution of di-8-ANEPPS in methanol was estimated from its molar extinction coefficient (ε) of 37,000 M^−1^cm^−1^ at 498 nm^46^. All other chemicals used were of the highest available purity. Water was purified through a Millipore (Bedford, MA) Milli-Q system and used throughout.

### Cell culture and cholesterol modulation of cells in culture

Chinese Hamster Ovary (CHO-K1) cells were maintained in DMEM/F-12 (1:1) supplemented with 2.4 g/l sodium bicarbonate, 10% FCS, and 60 μg/ml penicillin, 50 μg/ml streptomycin, 50 μg/ml gentamycin sulfate (complete DMEM) in a humidified atmosphere with 5% CO_2_ at 37 °C. Stock solution of lovastatin was prepared as described previously^90^. Cells were grown for 24 h in complete DMEM and then treated with increasing concentration of lovastatin for 48 h in complete DMEM. Control cells were grown under similar conditions in the absence of lovastatin. Acute cholesterol depletion was carried out using MβCD as described previously^34^. Briefly, cells were grown for 3 days followed by incubation in serum-free DMEM for 3 h at 37 °C. Cholesterol depletion was carried out by treating cells with increasing concentration of MβCD in serum-free DMEM for 30 min at 37 °C, followed by washing with PBS, pH 7.4 buffer. Replenishment of cholesterol to MβCD or lovastatin-treated cells was carried out as decribed previosly^81^.

### MTT viability assay

Viability of cells upon cholesterol depletion was assessed using MTT assay as described earlier^91^.

### Cell membrane preparation

Cell membranes were prepared as described previously^92^. Briefly, confluent cells were harvested by treatment with ice-cold buffer containing 10 mM Tris, 5 mM EDTA, 0.1 mM PMSF, pH 7.4. Cells were then homogenized for 10 s at 4 °C at maximum speed with a Polytron homogenizer. The cell lysate was centrifuged at 500 x g for 10 min at 4 °C and the resulting post-nuclear supernatant was centrifuged at 40,000 × g for 30 min at 4 °C. The pellet obtained was suspended in 50 mM Tris buffer, pH 7.4, flash frozen in liquid nitrogen and stored at −80 °C till further use. The total protein concentration in the isolated membranes was determined using BCA assay^93^.

### Estimation of cholesterol and phospholipid contents

Cholesterol content in cell membranes was estimated using Amplex Red cholesterol assay kit^94^. Total phospholipid content of membranes was determined subsequent to digestion with perchloric acid using Na_2_HPO_4_ as standard^95^. DMPC was used as an internal standard to assess lipid digestion. Samples without perchloric acid digestion produced negligible readings.

### Di-8-ANEPPS labeling of CHO-K1 cells

Membrane dipole potential measurements were carried out by dual wavelength ratiometric approach using voltage sensitive fluorescence probe di-8-ANEPPS^26–30^. Briefly, cells were plated at a density of ~10^4^ on glass cover slips and grown in complete DMEM. Following lovastatin or MβCD treatment, cells were washed with PBS and stained with 1 μM di-8-ANEPPS in serum-free DMEM for 30 min at 4 °C. Cells were then washed, fixed with 3.5% (v/v) formaldehyde for 10 min and mounted.

### Ratiometric fluorescence imaging

All images were acquired on an inverted Zeiss LSM 510 Meta confocal microscope (Jena, Germany) with a 63×/1.4 NA oil immersion objective under 1 airy condition. Di-8-ANEPPS labeled CHO-K1 cells were imaged using two excitation wavelengths (458 and 514 nm, corresponding to Argon laser lines) with a 650–710 nm emission band pass. The choice of the emission wavelength at the red edge of the fluorescence spectrum has previously been shown to rule out membrane fluidity effects^27^. Fluorescence intensity of images was corrected for the laser power at two different excitation wavelengths (458 and 514 nm). The fluorescence intensity ratio (R), defined as the ratio of fluorescence intensities at an excitation wavelength of 458 nm to that at 514 nm (650–710 nm emission band pass in both cases) was calculated using ImageJ (NIH, Bethesda, MD). A background intensity image was subtracted from the data for each image.

### Statistical analysis

Significance levels were estimated using Student’s two-tailed unpaired *t*-test using Graphpad Prism version 4.0 (San Diego, CA). The correlation between fluorescence intensity ratio and membrane cholesterol content was analyzed using the same software. Plots were generated using Microcal Origin version 6.0 (OriginLab, Northampton, MA).

## Electronic supplementary material

Supplementary Information

## References

[1] Bloch KE (1983). Sterol structure and membrane function. Crit. Rev. Biochem. Mol. Biol..

[2] Nes WD (2011). Biosynthesis of cholesterol and other sterols. Chem. Rev..

[3] Simons K, Ikonen E (2000). How cells handle cholesterol. Science.

[4] Mouritsen OG, Zuckermann MJ (2004). What’s so special about cholesterol?. Lipids.

[5] Krause MR, Regen SL (2014). The structural role of cholesterol in cell membranes: from condensed bilayers to lipid rafts. Acc. Chem. Res..

[6] Arora A, Raghuraman H, Chattopadhyay A (2004). Influence of cholesterol and ergosterol on membrane dynamics: a fluorescence approach. Biochem. Biophys. Res. Commun..

[7] Subczynski WK, Wisniewska A, Yin J-J, Hyde JS, Kusumi A (1994). Hydrophobic barriers of lipid bilayer membranes formed by reduction of water penetration by alkyl chain unsaturation and cholesterol. Biochemistry.

[8] Parasassi T, Di Stefano M, Loiero M, Ravagnan G, Gratton E (1994). Cholesterol modifies water concentration and dynamics in phospholipid bilayers: a fluorescence study using Laurdan probe. Biophys. J..

[9] Nezil FA, Bloom M (1992). Combined influence of cholesterol and synthetic amphiphilic peptides upon bilayer thickness in model membranes. Biophys. J..

[10] Liscum L, Underwood KW (1995). Intracellular cholesterol transport and compartmentation. J. Biol.Chem..

[11] Schroeder F, Woodford JK, Kavecansky J, Wood WG, Joiner C (1995). Cholesterol domains in biological membranes. Mol. Membr. Biol..

[12] Simons K, Ikonen E (1997). Functional rafts in cell membranes. Nature.

[13] Xu X, London E (2000). The effect of sterol structure on membrane lipid domains reveals how cholesterol can induce lipid domain formation. Biochemistry.

[14] Chaudhuri A, Chattopadhyay A (2011). Transbilayer organization of membrane cholesterol at low concentrations: implications in health and disease. Biochim. Biophys. Acta.

[15] Simons K, van Meer G (1988). Lipid sorting in epithelial cells. Biochemistry.

[16] Simons K, Toomre D (2000). Lipid rafts and signal transduction. Nat. Rev. Mol. Cell Biol..

[17] Pucadyil TJ, Chattopadhyay A (2007). Cholesterol: a potential therapeutic target in *Leishmania* infection?. Trends Parasitol..

[18] Kumar GA, Jafurulla M, Chattopadhyay A (2016). The membrane as the gatekeeper of infection: cholesterol in host-pathogen interaction. Chem. Phys. Lipids.

[19] Brockman H (1994). Dipole potential of lipid membranes. Chem. Phys. Lipids.

[20] Clarke RJ (2001). The dipole potential of phospholipid membranes and methods for its detection. Adv. Colloid Interface Sci..

[21] O’Shea P (2005). Physical landscapes in biological membranes: physico-chemical terrains for spatio-temporal control of biomolecular interactions and behaviour. Philos. Trans. A. Math. Phys. Eng. Sci..

[22] Wang L (2012). Measurements and implications of the membrane dipole potential. Annu. Rev. Biochem..

[23] Jewell SA, Petrov PG, Winlove CP (2013). The effect of oxidative stress on the membrane dipole potential of human red blood cells. Biochim. Biophys. Acta.

[24] Sarkar P, Chattopadhyay A (2015). Dipolar rearrangement during micellization explored using a potential-sensitive fluorescent probe. Chem. Phys. Lipids.

[25] Singh P, Haldar S, Chattopadhyay A (2013). Differential effect of sterols on dipole potential in hippocampal membranes: implications for receptor function. Biochim. Biophys. Acta.

[26] Gross E, Bedlack RS, Loew LM (1994). Dual-wavelength ratiometric fluorescence measurement of the membrane dipole potential. Biophys. J..

[27] Clarke RJ, Kane DJ (1997). Optical detection of membrane dipole potential: avoidance of fluidity and dye-induced effects. Biochim. Biophys. Acta.

[28] Starke-Peterkovic T, Turner N, Else PL, Clarke RJ (2005). Electric field strength of membrane lipids from vertebrate species: membrane lipid composition and Na^+^-K^+^-ATPase molecular activity. Am. J. Physiol. Regul. Integr. Comp. Physiol..

[29] Starke-Peterkovic T (2006). Cholesterol effect on the dipole potential of lipid membranes. Biophys. J..

[30] Haldar S, Kanaparthi RK, Samanta A, Chattopadhyay A (2012). Differential effect of cholesterol and its biosynthetic precursors on membrane dipole potential. Biophys. J..

[31] Bandari S, Chakraborty H, Covey DF, Chattopadhyay A (2014). Membrane dipole potential is sensitive to cholesterol stereospecificity: implications for receptor function. Chem. Phys. Lipids.

[32] Pucadyil TJ, Chattopadhyay A (2006). Role of cholesterol in the function and organization of G-protein coupled receptors. Prog. Lipid Res..

[33] Paila YD, Chattopadhyay A (2010). Membrane cholesterol in the function and organization of G-protein coupled receptors. Subcell. Biochem..

[34] Pucadyil TJ, Chattopadhyay A (2007). Cholesterol depletion induces dynamic confinement of the G-protein coupled serotonin_1A_ receptor in the plasma membrane of living cells. Biochim. Biophys. Acta.

[35] Pucadyil TJ, Chattopadhyay A (2004). Cholesterol modulates ligand binding and G-protein coupling to serotonin_1A_ receptors from bovine hippocampus. Biochim. Biophys. Acta.

[36] Paila YD, Murty MRVS, Vairamani M, Chattopadhyay A (2008). Signaling by the human serotonin_1A_ receptor is impaired in cellular model of Smith-Lemli-Opitzsyndrome. Biochim. Biophys. Acta.

[37] Shrivastava S, Pucadyil TJ, Paila YD, Ganguly S, Chattopadhyay A (2010). Chronic cholesterol depletion using statin impairs the function and dynamics of human serotonin_1A_ receptors. Biochemistry.

[38] Zidovetzki R, Levitan I (2007). Use of cyclodextrins to manipulate plasma membrane cholesterol content: evidence, misconceptions and control strategies. Biochim. Biophys. Acta.

[39] Mahammad S, Parmryd I (2015). Cholesterol depletion using methyl-β-cyclodextrin. Methods Mol. Biol..

[40] Kiss T (2010). Evaluation of the cytotoxicity of β-cyclodextrin derivatives: evidence for the role of cholesterol extraction. Eur. J. Pharm. Sci..

[41] Istvan ES, Deisenhofer J (2001). Structural mechanism for statin inhibition of HMG-CoA reductase. Science.

[42] Sirtori CR (2014). The pharmacology of statins. Pharmacol. Res..

[43] Schlyer S, Horuk R (2006). I want a new drug: G-protein-coupled receptors in drug development. Drug Discov. Today.

[44] Zheng H (2012). Palmitoylation and membrane cholesterol stabilize μ-opioid receptor homodimerization and G protein coupling. BMC Cell Biol..

[45] Loew LM, Scully S, Simpson L, Waggoner AS (1979). Evidence for a charge-shift electrochromic mechanism in a probe of membrane potential. Nature.

[46] Le Goff G, Vitha MF, Clarke RJ (2007). Orientational polarisability of lipid membrane surfaces. Biochim. Biophys. Acta.

[47] Loew LM (1996). Potentiometric dyes: imaging electrical activity of cell membranes. Pure Appl. Chem.

[48] Robinson D, Besley NA, O’shea P, Hirst JD (2011). Di-8-ANEPPS emission spectra in phospholipid/cholesterol membranes: a theoretical study. J. Phys. Chem. B.

[49] Burger K, Gimpl G, Fahrenholz F (2000). Regulation of receptor function by cholesterol. Cell. Mol. Life Sci..

[50] Chini B, Parenti M (2009). G-protein-coupled receptors, cholesterol and palmitoylation: facts about fats. J. Mol. Endocrinol..

[51] Paila YD, Tiwari S, Chattopadhyay A (2009). Are specific nonannular cholesterol binding sites present in G-protein coupled receptors?. Biochim. Biophys. Acta.

[52] Oates J, Watts A (2011). Uncovering the intimate relationship between lipids, cholesterol and GPCR activation. Curr. Opin. Struct. Biol..

[53] Lee AG (2011). Lipid-protein interactions. Biochem. Soc. Trans..

[54] Fantini J, Barrantes FJ (2013). How cholesterol interacts with membrane proteins: an exploration of cholesterol-binding sites including CRAC, CARC, and tilted domains. Front. Physiol..

[55] Jafurulla M, Chattopadhyay A (2013). Membrane lipids in the function of serotonin and adrenergic receptors. Curr. Med. Chem..

[56] Paila YD, Chattopadhyay A (2009). The function of G-protein coupled receptors and membrane cholesterol: specific or general interaction?. Glycoconj. J..

[57] Lee AG (2011). Biological membranes: the importance of molecular detail. Trends Biochem. Sci..

[58] Klein U, Gimpl G, Fahrenholz F (1995). Alteration of the myometrial plasma membrane cholesterol content with *β*-cyclodextrin modulates the binding affinity of the oxytocin receptor. Biochemistry.

[59] Bari M, Battista N, Fezza F, Finazzi-Agrò A, Maccarrone M (2005). Lipid rafts control signaling of type-1 cannabinoid receptors in neuronal cells. Implications for anandamide-induced apoptosis. J. Biol. Chem..

[60] Lam RS, Nahirney D, Duszyk M (2009). Cholesterol-dependent regulation of adenosine A_2A_ receptor-mediated anion secretion in colon epithelial cells. Exp. Cell Res..

[61] Emery AC, Liu X-H, Xu W, Eiden MV, Eiden LE (2015). Cyclic adenosine 3′,5′-monophosphate elevation and biological signaling through a secretin family G_s_-coupled G protein-coupled receptor are restricted to a single adenylate cyclase isoform. Mol. Pharmacol..

[62] Pydi SP (2016). Cholesterol modulates bitter taste receptor function. Biochim. Biophys. Acta.

[63] Sjögren B, Hamblin MW, Svenningsson P (2006). Cholesterol depletion reduces serotonin binding and signaling via human 5-HT_7(a)_ receptors. Eur. J. Pharmacol..

[64] Hu C-H (2010). Effects of simvastatin and 6-hydroxydopamine on histaminergic H1 receptor binding density in rat brains. Prog. Neuropsychopharmacol. Biol. Psychiatry.

[65] Banfi C (2011). Statins prevent tissue factor induction by protease-activated receptors 1 and 2 in human umbilical vein endothelial cells *in vitro*. J. Thromb. Haemost..

[66] Ilangumaran S, Hoessli DC (1998). Effects of cholesterol depletion by cyclodextrin on the sphingolipid microdomains of the plasma membrane. Biochem. J..

[67] Kwik J (2003). Membrane cholesterol, lateral mobility, and the phosphatidylinositol 4,5-bisphosphate-dependent organization of cell actin. Proc. Natl. Acad. Sci. USA.

[68] Goodwin JS, Drake KR, Remmert CL, Kenworthy AK (2005). Ras diffusion is sensitive to plasma membrane viscosity. Biophys. J..

[69] Breusegem SY (2005). Acute and chronic changes in cholesterol modulate Na-P_i_ cotransport activity in OK cells. Am. J. Physiol. Renal Physiol.

[70] Shvartsman DE, Gutman O, Tietz A, Henis YI (2006). Cyclodextrins but not compactin inhibit the lateral diffusion of membrane proteins independent of cholesterol. Traffic.

[71] Cheng J, Ohsaki Y, Tauchi-Sato K, Fujita A, Fujimoto T (2006). Cholesterol depletion induces autophagy. Biochem. Biophys. Res. Commun..

[72] Paila YD, Kombrabail M, Krishnamoorthy G, Chattopadhyay A (2011). Oligomerization of the serotonin_1A_ receptor in live cells: a time-resolved fluorescence anisotropy approach. J. Phys. Chem. B.

[73] Mukherjee S, Maxfield FR (2004). Membrane domains. Annu. Rev. Cell Dev. Biol..

[74] Lingwood D, Simons K (2010). Lipid rafts as a membrane-organizing principle. Science.

[75] Kilsdonk EPC (1995). Cellular cholesterol efflux mediated by cyclodextrins. J. Biol. Chem..

[76] Yancey PG (1996). Cellular cholesterol efflux mediated by cyclodextrins. Demonstation of kinetic pools and mechanism of efflux. J. Biol. Chem..

[77] Sanchez SA, Gunther G, Tricerri MA, Gratton E (2011). Methyl-β-cyclodextrins preferentially remove cholesterol from the liquid disordered phase in giant unilamellar vesicles. J. Membr. Biol..

[78] López CA, de Vries AH, Marrink SJ (2013). Computational microscopy of cyclodextrin mediated cholesterol extraction from lipid model membranes. Sci. Rep..

[79] Mahammad S, Parmryd I (2008). Cholesterol homeostasis in T cells. Methyl-β-cyclodextrin treatment results in equal loss of cholesterol from Triton X-100 soluble and insoluble fractions. Biochim. Biophys. Acta.

[80] Liao JK, Laufs U (2005). Pleiotropic effects of statins. Annu. Rev. Pharmacol. Toxicol..

[81] Singh P, Saxena R, Srinivas G, Pande G, Chattopadhyay A (2013). Cholesterol biosynthesis and homeostasis in regulation of the cell cycle. PLoS One.

[82] Starke-Peterkovic T, Clarke RJ (2009). Effect of headgroup on the dipole potential of phospholipid vesicles. Eur. Biophys. J..

[83] Duffin RL, Garrett MP, Flake KB, Durrant JD, Busath DD (2003). Modulation of lipid bilayer interfacial dipole potential by phloretin, RH421, and 6-ketocholestanol as probed by gramicidin channel conductance. Langmuir.

[84] Clarke RJ (2015). Dipole-potential-mediated effects on ion pump kinetics. Biophys. J..

[85] Richens JL, Lane JS, Bramble JP, O’Shea P (2015). The electrical interplay between proteins and lipids in membranes. Biochim. Biophys. Acta.

[86] Cladera J, O’Shea P (1998). Intramembrane molecular dipoles affect the membrane insertion and folding of a model amphiphilic peptide. Biophys. J..

[87] Chaudhuri A, Chattopadhyay A (2014). Lipid binding specificity of bovine α-lactalbumin: a multidimensional approach. Biochim. Biophys. Acta.

[88] Sarkar P, Chattopadhyay A (2016). Micellar dipole potential is sensitive to sphere-to-rod transition. Chem. Phys. Lipids.

[89] Menge T, Hartung H-P, Stüve O (2005). Statins — a cure-all for the brain?. Nat. Rev. Neurosci..

[90] Keyomarsi K, Sandoval L, Band V, Pardee AB (1991). Synchronization of tumor and normal cells from G1 to multiple cell cycles by lovastatin. Cancer Res..

[91] Roy S, Kumar GA, Jafurulla M, Mandal C, Chattopadhyay A (2014). Integrity of the actin cytoskeleton of host macrophages is essential for *Leishmania donovani* infection. Biochim. Biophys. Acta.

[92] Kalipatnapu S, Pucadyil TJ, Harikumar KG, Chattopadhyay A (2004). Ligand binding characteristics of the human serotonin_1A_ receptor heterologously expressed in CHO cells. Biosci. Rep..

[93] Smith PK (1985). Measurement of protein using bicinchoninic acid. Anal. Biochem..

[94] Amundson DM, Zhou M (1999). Fluorometric method for the enzymatic determination of cholesterol. J. Biochem. Biophys. Methods.

[95] McClare CWF (1971). An accurate and convenient organic phosphorus assay. Anal. Biochem..

