# Supplementary material for: Differential Membrane Dipolar Orientation Induced by Acute and Chronic Cholesterol Depletion
Source: Sci Rep. 2017 Jun 30;7:4484. doi: 10.1038/s41598-017-04769-4 (PMC5493612; doi:10.1038/s41598-017-04769-4)
Supplement: Supplementary file 1 — Supplementary Information [file 41598_2017_4769_MOESM1_ESM.pdf]

Supporting Information

Differential Membrane Dipolar Orientation Induced by Acute and Chronic Cholesterol Depletion

Parijat Sarkar[†], Hirak Chakraborty[†,#], and Amitabha Chattopadhyay[†,*]

[†]CSIR-Centre for Cellular and Molecular Biology, Uppal Road, Hyderabad 500 007, India; [#]School of Chemistry, Sambalpur University, Jyoti Vihar, Burla, Odisha 768 019, India

*To whom correspondence should be addressed: Phone: +91-40-2719-2578,
Fax: +91-40-2716-0311, E-mail: amit@ccmb.res.in

Figure S1
Sarkar *et al.*

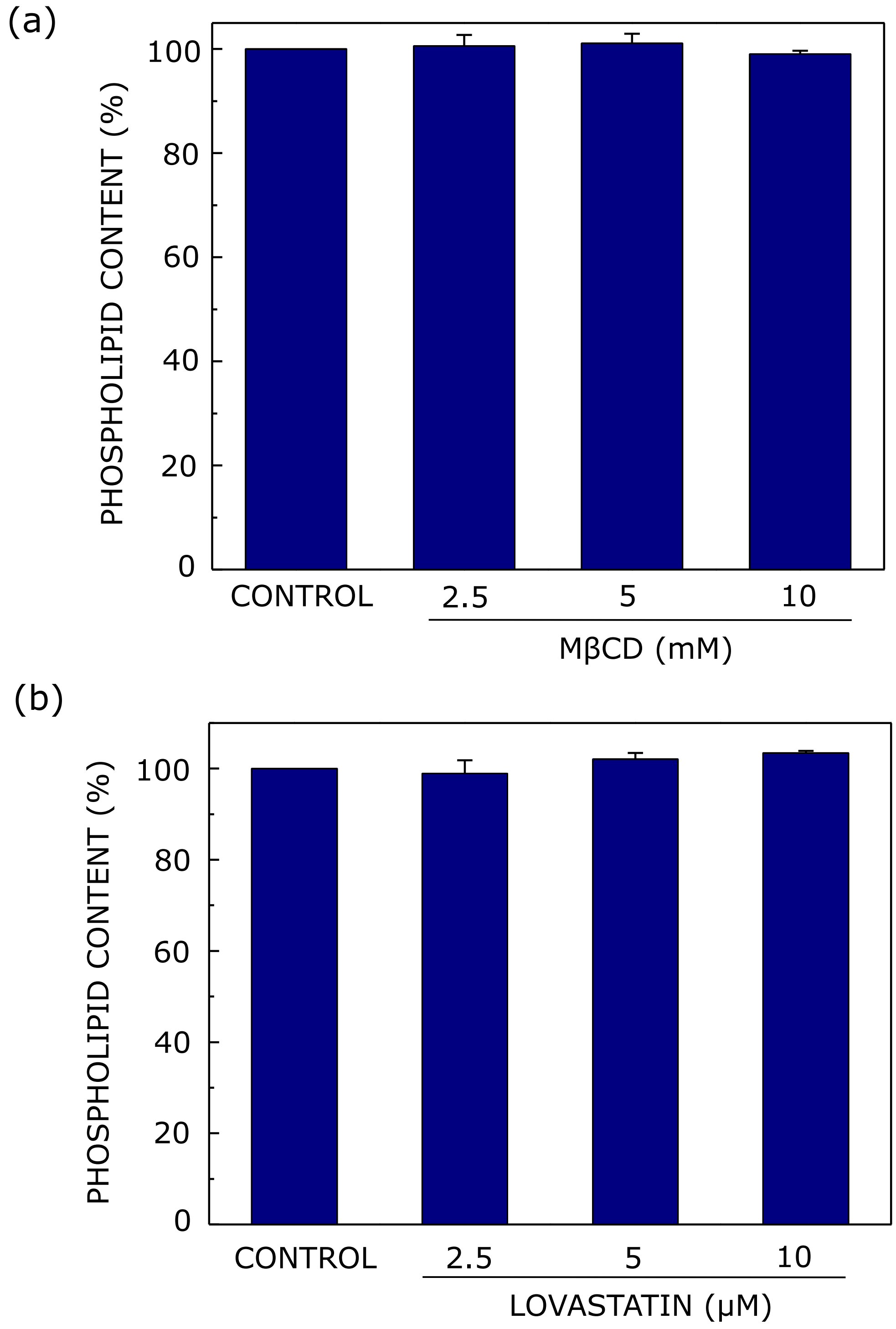

Figure S1. Membrane phospholipid content remains invariant upon acute or chronic cholesterol depletion. Effect of increasing concentration of (a) MβCD and (b) lovastatin on phospholipid content of CHO-K1 cell membranes. Values are expressed as percentage of phospholipid content for cell membranes in the absence of MβCD or lovastatin treatment. Data represent means ± S.E. of at least three independent measurements. See Methods for other details.

Figure S2
Sarkar *et al.*

(a)

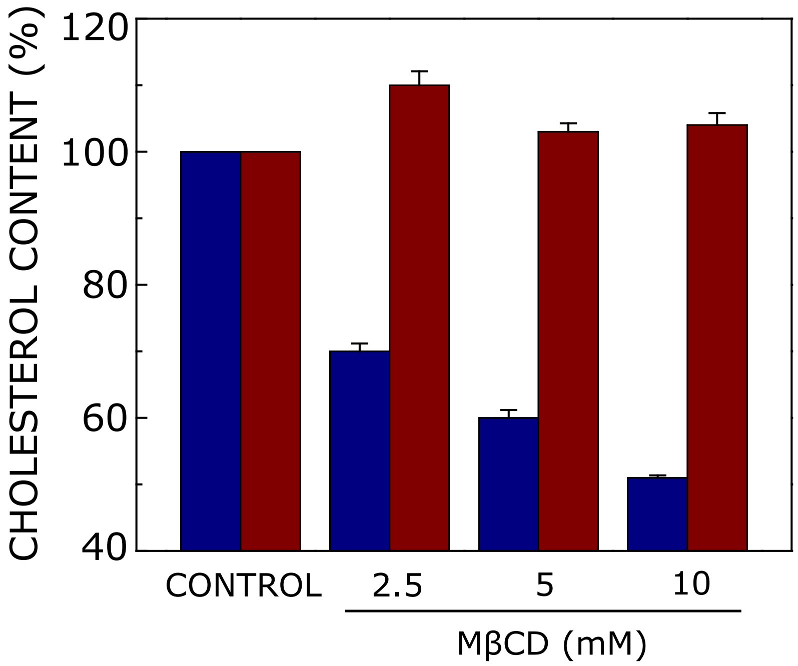

(b)

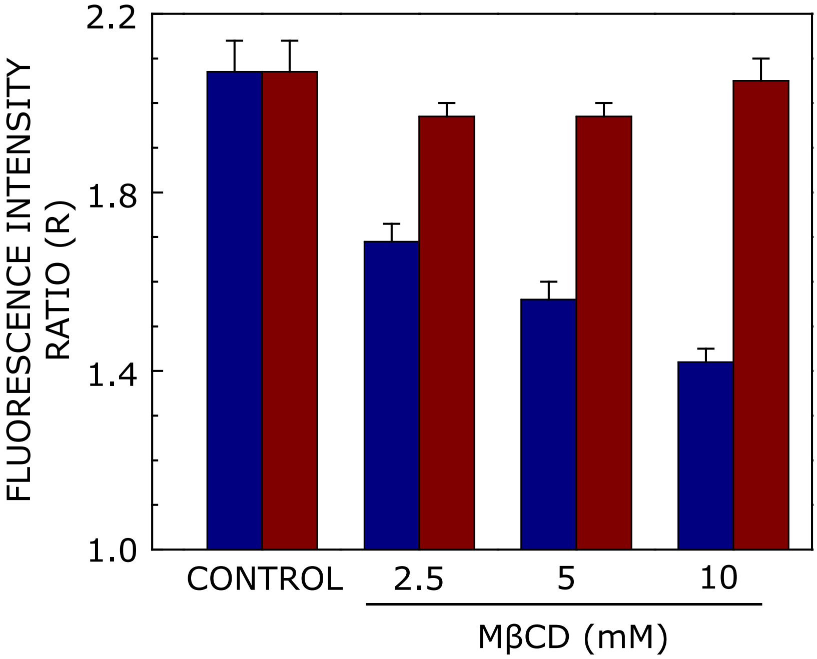

Figure S2. The change in membrane dipole potential upon acute cholesterol depletion could be reversed upon replenishment with cholesterol. Cholesterol content (a) and fluorerscence intensity ratio, R (b) for cholesterol-depleted (blue bars) and cholesterol-replenished (maroon bars) are shown. See Methods for other details.

Figure S3
Sarkar *et al.*

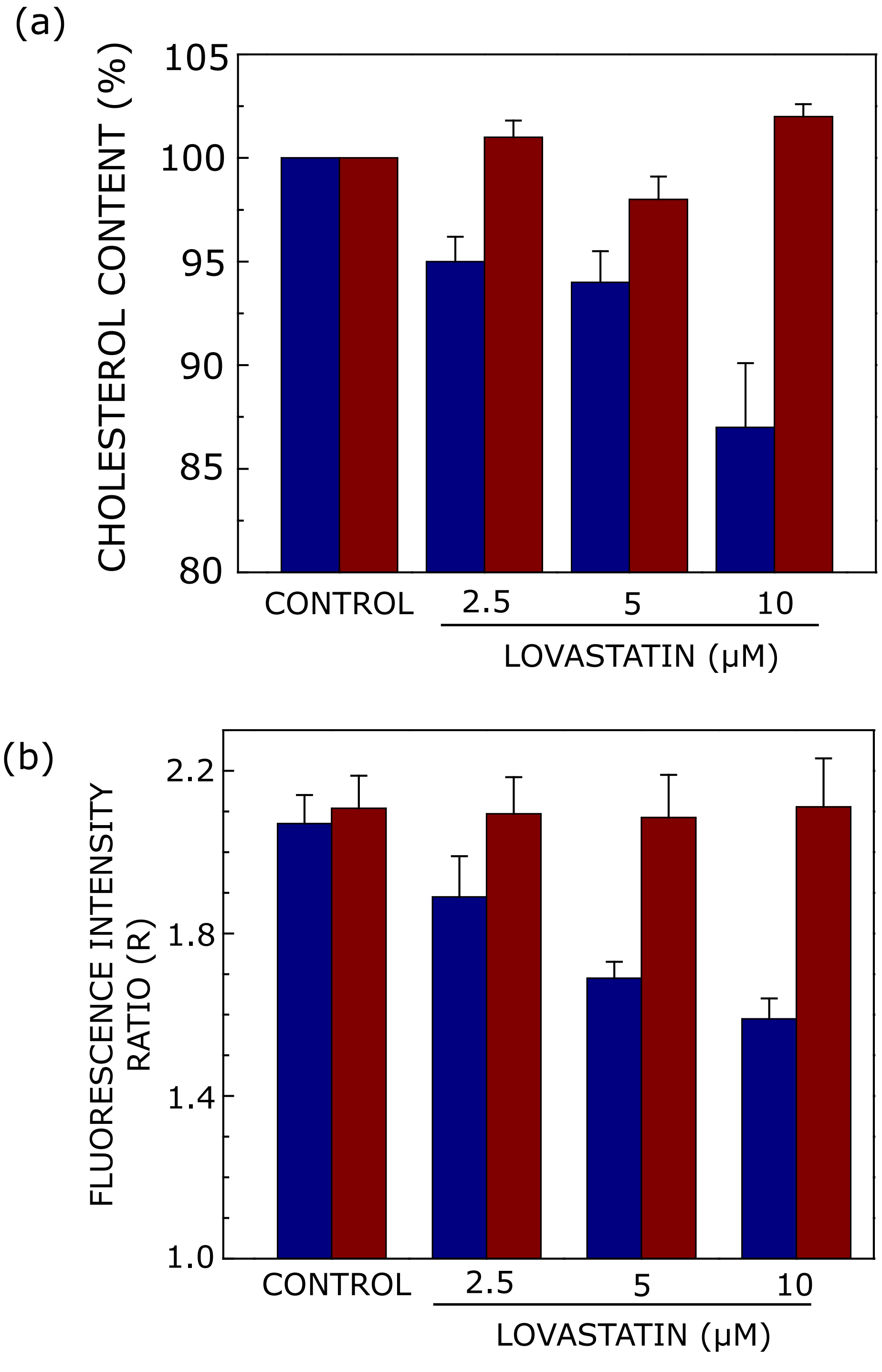

Figure S3. The change in membrane dipole potential upon chronic cholesterol depletion could be reversed upon replenishment with cholesterol. Cholesterol content (a) and fluorerscence intensity ratio, R (b) for cholesterol-depleted (blue bars) and cholesterol-replenished (maroon bars) are shown. See Methods for other details.
